# The Conservation Status of the Barbary Macaque (*Macaca sylvanus*) in Algeria: Population Estimates and Human Impacts

**DOI:** 10.3390/ani15131860

**Published:** 2025-06-24

**Authors:** Mourad Boumenir, Fany Brotcorne, Jean-Luc Hornick, Mokrane Iguer-Ouada, Nassim Moula

**Affiliations:** 1 FARAH Research Unit, Department of Veterinary Management of Animal Resources, Faculty of Veterinary Medicine, University of Liege, 4000 Liege, Belgium; mourad.boumenir@doct.uliege.be (M.B.); jlhornick@uliege.be (J.-L.H.); 2 SPHERES Research Unit, Behaviour Biology Unit, Department of Biology, Ecology, and Evolution, Faculty of Sciences, University of Liège, 4020 Liege, Belgium; fbrotcorne@uliege.be; 3 Associated Laboratory in Marine Ecosystems and Aquaculture, Department of Biological Sciences of the Environment, Faculty of Nature and Life Sciences, University of Bejaia, Bejaia 06000, Algeria; imokrane@gmail.com; 4 GIGA Animal Facilities, University of Liege, 4000 Liege, Belgium

**Keywords:** *Macaca sylvanus*, Algeria, primate conservation, protected areas management, anthropogenic pressures

## Abstract

**Simple Summary:**

The Barbary macaque, the only North African primate, is currently Endangered. The present review aims to provide a comprehensive overview of the current state of knowledge on the Barbary macaque in Algeria by compiling the most recent scientific and institutional data to better understand the distribution of this primate and the threats it faces. We estimated that more than 9000 macaques are found in Algeria, although comprehensive surveys are still lacking in many regions. Habitat loss, overgrazing, road traffic, illegal trade, and tourism are the main threats affecting macaque populations. The findings from this review reveal a pressing need to strengthen protection measures for this flagship primate species, including the creation of protected areas in the Guerrouche and Akfadou forests, the implementation of enforceable legislation to address illegal trade, and the installation of road signs and speed bumps to reduce roadkill.

**Abstract:**

The Barbary macaque (*Macaca sylvanus*), the only African macaque and an endangered species, faces significant conservation challenges. By compiling both published and unpublished data, this review aims to provide a comprehensive synthesis of the current state of knowledge on the Barbary macaque in Algeria, with a specific focus on (1) geographic distribution and population estimates, and (2) anthropogenic threats. We reviewed 409 studies and identified 41 relevant to the Algerian context. Our findings update population estimates, revealing over 9000 individuals across national parks such as Djurdjura, Gouraya, and Chréa. We also document new presence data of the species in Skikda and Jijel. Finally, an analysis of the available studies on the impact of anthropogenic activities on the species in Algeria shows that the primary factors affecting macaques include habitat loss, overgrazing, illegal trade, road mortality, and tourism-related disease transmission. While species monitoring has improved, many forest regions remain understudied. We call for expanded systematic research and conservation efforts, particularly in unsurveyed habitats such as the Akfadou and Guerrouche forests and Babors-Tababort National Park. By integrating diverse data sources, this review supports the need for evidence-based conservation of *M. sylvanus* in Algeria and highlights its critical role in North African biodiversity.

## 1. Introduction

The Barbary macaque (*Macaca sylvanus*—Linnaeus, 1758), also known as the Berber macaque or “Magot,” is the only non-human primate species native to North Africa and the single macaque species living outside Asia [1]. As a bioindicator of forest health in this region, *M*. *sylvanus* plays a key ecological role in forest ecosystems, particularly in cedar and oak forests in North Africa. It contributes to forest maintenance and regeneration via seed dispersal of some tree species, and serves as an umbrella species for habitat conservation [2].

*Macaca. sylvanus* inhabits various types of habitats including cedar (*Cedrus atlantica*—(Endl.) Manetti ex Carrière, 1855), holm oak (*Quercus ilex*—Linnaeus, 1753), cork oak (*Q. suber*—Linnaeus, 1753), and mixed oak forests [3,4,5]. These natural habitats ensure a consistent availability of food throughout the year. However, cedar forests appear to be the most ecologically favorable habitat for this species [3,6,7,8]. Barbary macaques are classified as primary consumers, relying on plant matter for their food, although they may occasionally supplement their diet with animal matter such as insects, mollusks, eggs and, during periods of food scarcity, even hunting rabbits and chicks [7,9,10].

Regarding its social structure, this tolerant macaques species typically live in multi-male, multi-female groups [11,12,13]. The mating system is polygamous, with an adult individual capable of mating with several partners during the same breeding season [14,15]. Female philopatry is observed in this species, with males migrating between groups during the breeding season to find new mates [16,17,18].

Historically widespread across the Mediterranean Basin, the current distribution of *M. sylvanus* is now restricted to fragmented forest regions in Algeria and Morocco, with a small introduced population on the Rock of Gibraltar [1]. The first global population estimates of the wild Barbary macaque date back nearly four decades ago, placing the total population at approximately 22,000 individuals across its entire range [1]. Around 16,500 occur in Morocco, primarily in the Middle Atlas Mountains, while between 5000 and 6200 individuals are estimated to occur in Algeria. The Algerian population was reported to be distributed across seven main localities: Djurdjura (1450–1750 individuals), Akfadou (1310–2100), Guerrouche (1500), Chiffa (300), Babors (300), Kherrata (200) and Pic des Singes (Béjaïa, 15–50) [1]. Recent population studies indicate that the wild Moroccan macaque populations are in continuous decline due to a combination of anthropogenic pressures, including habitat loss, illegal trade, overgrazing, and increasing interaction with human populations [19,20,21,22]. Consequently, the Barbary macaque is currently classified as Endangered by the International Union for Conservation of Nature (IUCN) [21,22,23], and is listed in Appendix I of CITES, which prohibits its trade. Additionally, national legislation in Algeria and Morocco designates it as a protected species, strictly prohibiting capture, hunting, possession, and sale.

The need for an updated and Algeria-focused review of the Barbary macaque is underscored by several conservation realities. Previous large-scale reviews have offered important contributions to the understanding of *M. sylvanus* biology, conservation, and management [1,24,25,26]. However, the distribution of the Barbary macaque in Algeria as reported in these reviews, largely relies on extrapolated data from Fa et al. [1] rather than from a recent census of individuals, which suggests that current populations may be underestimated or overestimated. Furthermore, most available studies from Algeria remain fragmented, unpublished, or difficult to obtain, such as master’s theses or internal national park reports. In addition, previous reviews [2,22,23,24] still refer to outdated geographic designations for the distribution of *M. sylvanus* in Algeria, including Grande Kabylie, Petite Kabylie, and Djurdjura. Many of the latter geographic areas have since been officially recognized as national parks or protected areas. Updating the terminology of occurrence areas is necessary to better reflect the current administrative classifications and enhance the precision of future conservation studies. Finally, previous reviews have largely relied on studies conducted in Morocco to characterize anthropogenic threats, which has led to a limited understanding of the specific pressures affecting Algerian populations and their conservation needs.

This review addresses these gaps by offering the first Algeria-focused comprehensive synthesis of available data on *M. sylvanus*. Specifically, it aims to explore the following: (1) Update macaque population estimates and distribution data, integrating both published and unpublished sources, including recent national park reports and student field surveys. (2) Identify and analyze major anthropogenic threats impacting macaque populations in Algeria. (3) Suggest research priorities and directions to support a more effective conservation strategy in Algeria. Through this analysis, we aim to deliver a region-specific, evidence-based assessment of the conservation status of *M. sylvanus* in Algeria, contributing to both academic discourse and field-based policy and management.

## 2. Material and Methods

### 2.1. Literature Search Strategy

This review followed a structured protocol to collect and evaluate existing information on *M. sylvanus* in Algeria, focusing on two main axes: (1) population size and distribution, and (2) anthropogenic pressures. A systematic search of the literature was conducted across four major academic databases: PubMed, CAB Abstracts, Academic Research Premier, and Google Scholar. The search covered all publications available prior to April 2025 and included peer-reviewed articles, grey literature, and institutional reports. We used the following search terms (in English or French) in the title field: “*Macaca sylvanus*”, “Barbary macaque*”, “Berber macaque*”, “Barbary ape*”. Boolean operators (AND/OR) were used to expand results. We did not apply language or date restrictions to ensure a broad dataset. To address the scarcity of peer-reviewed publications specific to Algeria, we expanded our search to include governmental and institutional sources: internal reports from the National Parks of Djurdjura, Gouraya, and Chréa; data shared by the Forest Conservation Directorate of Béjaia; unpublished academic theses (master’s and doctoral level), reviewed manually for methodological consistency. These documents provided critical information on recent censuses, macaque sightings, and forest conditions unpublished in scientific journals. To avoid redundancy, duplicate entries were removed using the Mendeley Desktop reference manager (version 1.19.8).

### 2.2. Inclusion and Exclusion Criteria

Following reference identification, we subsequently included studies that met the following criteria: (1) study including data on “*M. sylvanus*” in Algeria and (2) provides data on population, distribution, or anthropogenic threats. We eliminated studies using the following exclusion criteria: (1) studies exclusively focused on Moroccan populations or captive macaques; (2) no specific data on Algeria; (3) reviews, book sections (i.e., did not offer additional data in relation to the research articles already included); (4) survey methodology unclears (i.e., missing or insufficient details on census and identification methods); (5) Theses already published as journal articles.

### 2.3. Census Methodology of Included Studies

Population estimates included in this review were based on direct counts conducted during field surveys using a standardized approach [27]. Individual macaques were identified based on morphological characteristics (e.g., coat color, facial patterns, testicular descent), following the age and sex classification criteria established by Ménard et al. [27]. Key individuals with distinctive features were used as reference points to differentiate between neighboring groups. Over three consecutive days, direct counts were performed through repeated observations during procession counts, in which a team of two or three observers recorded the number of individuals as the macaques traveled collectively across open areas such as roads or trails (Table 1). 

### 2.4. Data Screening

**Figure 1:**
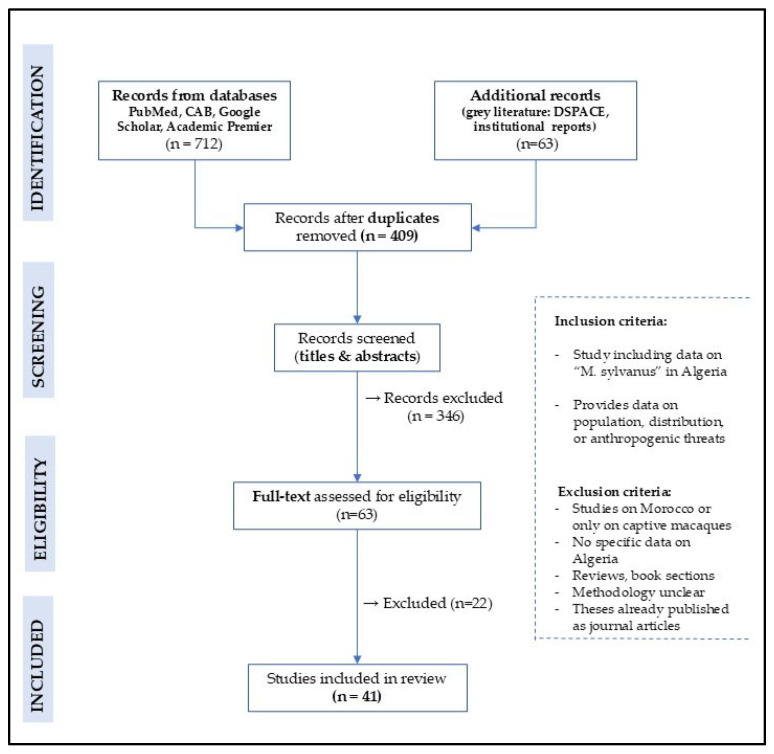
PRISMA diagram of records included in the present review on Barbary macaques in Algeria.

All records were first screened by title and abstract, and then, when necessary, full-text analysis was performed. A total of 409 unique references were retained after removing duplicates. A total of 41 studies were included in the present review, including 24 journal articles, 13 theses, 3 institutional reports and 1 conference paper. The selection process is summarized in Figure 1 using a PRISMA-style reporting format. 

### 2.5. Mapping and Spatial Analysis

**Figure 2:**
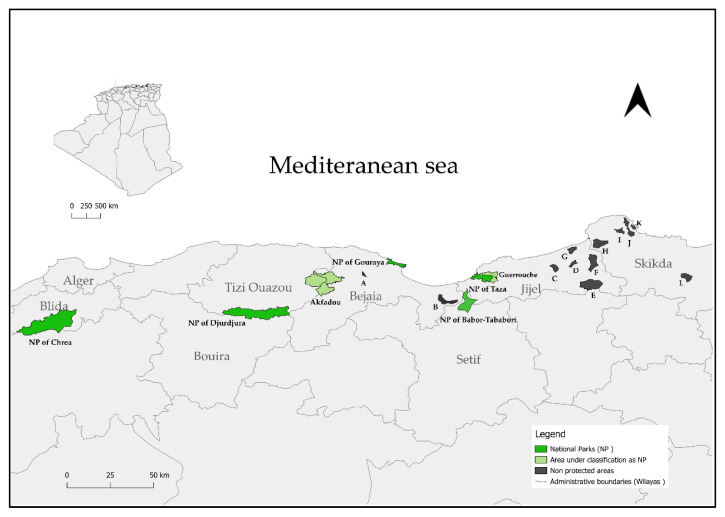
Current distribution map of the Barbary macaque (*Macaca sylvanus*) in Algeria. Colored polygons (green = protected areas; black = non-protected areas) represent areas where populations of the species are currently present, based on recent field data and institutional reports. Letters refer to specific localities: in Béjaïa: A—Bourbaatache (El-Kseur), B—Kherrata; in Jijel: C—Bordj Tahar, D—El-Ancer, E—Sidi Marouf and Aïn Lahmame, F—El Milia, G—Khier Oued Adjoul; in Skikda: H—Oued Z’hour, I—Zitounia, J—Cheraia, K—Collo, L—Salah Bouchaour.

To visualize the distribution of *M. sylvanus*, we created an updated distribution map using QGIS v3.36.0 [32]. Data layers included: (1) IUCN Red List spatial data “(https://www.iucnredlist.org/resources/spatial-data-download (accessed on 15 April 2025)”, (2) GBIF occurrence records “(https://www.gbif.org (accessed on 15 April 2025)”, (3) Aadministrative boundaries from GADM v4.0 “(https://gadm.org (accessed on 15 April 2025)”, and (4) Ddigitized forest coverage maps and park delimitations from official Alge-rian sources. Manual coordinates were also extracted from field reports where possible. The map presented in Figure 2 offers a visual overview of known populations and survey coverage.

To ensure greater geographic precision and to align with modern conservation frameworks, this review systematically adopts the updated nomenclature, reflecting the current legal and ecological status of these protected areas. 

### 2.6. Ethical Considerations

As this study is a narrative review, no new fieldwork or experimentation involving animals was conducted. All institutional documents and reports used were public or shared with permission.

## 3. Results

### 3.1. Distribution and Recent Population Estimates of Macaca sylvanus in Algeria

The present review integrates recent studies based on direct counts of individuals [33], providing direct population estimates and reducing the biases inherent in earlier extrapolation-based methods applied to larger areas [1]. Between 2012 and 2023, several localized field surveys and national park management reports provided updated information on the status of *M. sylvanus* populations in northern Algeria (Table 2 and Figure 2). Although repeated counts were conducted on the same groups, the authors reported only the mean of these counts, without providing uncertainty intervals (minimum–maximum). Moreover, previous descriptions of the distribution of *M*. *sylvanus* often referred to broad historical geographical regions, without precise alignment with current administrative classifications. Since the 1980s, several of these forested massifs, notably Djurdjura, Taza, Gouraya, Chréa, and the Babors-Tababort, have been officially designated as national parks [34].

Our review demonstrates that the National Park of Djurdjura, remains the principal refuge of the species (Figure 2). Indeed, the population survey conducted in Djurdjura National Park (185.5 km^2^) recorded 102 social groups totaling around 4645 individuals distributed across various sectors [31]. In Gouraya National Park (Béjaïa), although the area is smaller (20.8 km^2^), 12 groups comprising approximately 315 individuals were recorded in 2016 [28]. Similarly, Chréa National Park (265.87 km^2^) is estimated to host around 630 individuals distributed across 14 groups [35]. In the National Park of Taza (Jijel, 62.35 km^2^), 19 groups were identified; however, only the anthropogenic groups located along the littoral areas (i.e., 6 groups) were subject to individual counts, totaling 252 macaques. The remaining groups that primarily colonize the Guerrouche Forest could not be properly counted [27,36] (Figure 2).

Though less intensive, surveys have also confirmed the presence of *M. sylvanus* in other regions. Outside the National Park of Taza, within the same Jijel region, seven additional groups were recorded, accounting for a total of 203 individuals [29,30]. Finally, smaller populations have been reported in the Skikda region (4 groups, 43 individuals) and in the Béjaïa region at El-Kseur (~30 individuals) [30].

Based on recent field surveys and historical estimates for regions that have not been recently surveyed, the total population of *M. sylvanus* in Algeria is estimated to range between 9599 and 10,389 individuals, with an approximate average of 9994 individuals. This figure includes around 6300 individuals directly counted between 2012 and 2023, and approximately 3300 individuals inferred from older estimates for regions such as Akfadou, Guerrouche, Babors, and Kherrata (Table 2).

### 3.2. Habitat Loss and Fragmentation

Habitat degradation is one of the most significant threats to *M. sylvanus* in Algeria. According to recent analyses, forest cover in northern Algeria has declined by over 64% in the past few decades, driven by deforestation, wildfires, illegal logging, and urban expansion [37,38]. Considering that Barbary macaque populations in Algeria are all confined in the north, the loss of forest cover is driving the fragmentation of this species habitat. Such fragmentation reduces genetic flow between the different populations, because functional connectivity between the various zones is greatly limited [39]. In Algeria, four studies have demonstrated that the remaining metapopulation, taken as a whole, still shows a marked genetic variability. In other words, the forest fragmentation in Algeria had not yet led to a reduction in genetic variability [17,18,40,41]. However, it has led to clear genetic isolation among the different subpopulations in this region [18,40,41]. It is important to note that a forest edge of more than one kilometer or the presence of human-dominated landscapes (habitations, agricultural zones, etc.) constitute a barrier to the movement of Barbary macaques and therefore disrupt the genetic flow between populations [42].

### 3.3. Overgrazing and Livestock Encroachment

It is commonly recognized that overgrazing has a direct or indirect influence on the Barbary macaque via four ecological factors: modified interspecific competition, increased predation, decreased availability of food resources, and habitat degradation [43,44,45,46,47,48]. In Morocco, overgrazing is likely associated with a dramatic decline in *M. sylvanus* populations in the Middle Atlas Mountains [20].

In Algeria, only three studies reported the impact of overgrazing on the Barbary macaque. According to [47], in the rocky ridges of Akfadou, where populations of Barbary macaques have been studied, overgrazing reduced the availability of water and food resources, exposing the macaques to attacks by dogs accompanying the herds. The same study indicates that overgrazing in the Djurdjura region was less pronounced and did not pose a risk to this species. More recently, a comparative study conducted at three sites with different degrees of grazing (two in the Atlas Mountains in Morocco and one in Djurdjura in Algeria) indicated that the herbaceous richness of the diet of *M. sylvanus* in Morocco is strongly altered by overgrazing [45]. A recent study conducted in the region of Djurdjura indicates a continuous growth in the size of livestock over the past few years [49]. The livestock population in the Aït Ouabane sector increased by more than 122% between 2004 and 2014. The same authors suggest that overgrazing is the main cause of the human-macaque conflict in this region. This suggests that Barbary macaque populations in this region may also be experiencing the pressure of overgrazing, similar to the Moroccan populations. These studies do not allow for definitive conclusions, and the quantitative impacts of overgrazing requires more in-depth investigations to enhance the conservation efforts for the Barbary macaque in Algeria.

### 3.4. Poaching and Illegal Trade

The trade of *M. sylvanus* and its use as a pet is a very old practice [50,51]. The earliest evidence of trade in these primates’ dates back to the Egyptian pharaohs, where Barbary macaques were found mummified in a pyramid [52]. Researchers have also identified, through osteology and genetic techniques, a juvenile Barbary macaque skeleton in Pompeii (Italy) dating back to Roman times [53]. These trading practices, which were previously considered legal, increased in the 1960s and 1970s [51]. As a result, the trade in this species has been one of the main causes of the extraordinary decline in its wild populations [20,46,54]. Today, this practice is considered illegal due to the species’ inclusion in the Appendix I of CITES.

In Algeria, only two studies have reported illegal trade in Barbary macaques. These studies collected online data from websites in Algeria involving the sale of Barbary macaque individuals. The results show the presence of eight advertisements for the sale of Barbary macaques on the Ouedkniss website, although the origin of the macaques was unknown. The age of the individuals offered for sale ranged from 3 months to 4 years. The selling price was between USD 450 and 600 [55,56]. These results highlight two major concerns. Firstly, the fact that there is only one study on this subject suggests a lack of prior attention to this issue in scientific research and conservation in Algeria. Secondly, the presence of such clearly illegal activity on online platforms calls for an urgent reinforcement of monitoring efforts to control this harmful business. The Barbary macaque is currently protected by legislation under Executive Decree No. 12–235 of 3 Rajab 1433 corresponding to 24 May 2012 establishing the list of protected non-domestic animal species in Algeria. This decree allows the authorities to confiscate animals found in the possession of individuals and to address fines against them. However, this legislative text does not provide for any repressive measures (sanctions, penalties) for trade at a national level. To this end, it is essential to consider the issue at the national scale to promote an appropriate legislative mechanism that would provide reinforced legal protection for this primate.

### 3.5. Road Mortality

**Figure 3:**
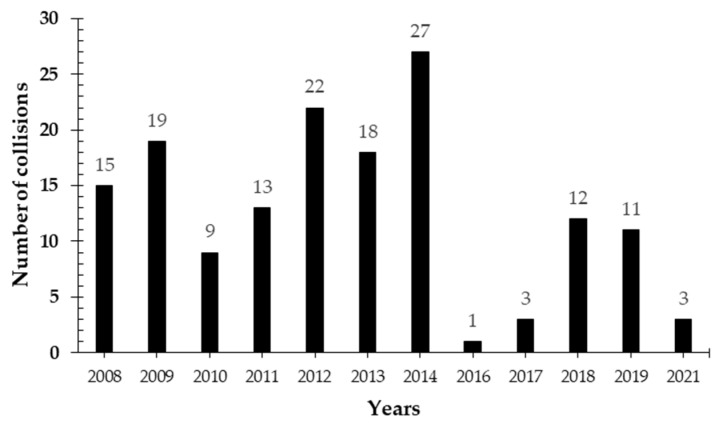
Collisions involving vehicles and *M. sylvanus* individuals in the Chréa National Park during the period 2008–2021 (according to the veterinary service of the National Park and [57]).

Expanding road networks across national parks and forest edges are a major source of macaque mortality, especially in Djurdjura and Chréa. Macaques often forage or cross roads, attracted by food left by tourists or simply due to fragmented forest layouts. In Chréa National Park, the park veterinary service and a recent study recorded over 150 macaque deaths from vehicle collisions between 2008 and 2021 (Figure 3) [57]. In Djurdjura, 23 roadkill cases were documented between 2017 and 2021 by the veterinary service, with 14 individuals found dead and 9 injured animals rescued and rehabilitated.

Roads not only cause mortality but also act as physical and behavioral barriers, disrupting group cohesion and movement patterns. Consequently, it can alter their ranging and dispersal behavior, causing macaques to avoid certain zones or become habituated to human presence, further increasing risks.

### 3.6. Tourism and Disease Transmission

Tourism associated with wildlife, particularly primates, is a growing economy that could have conservation benefits when implemented properly [58]. However, it is essential to assess the impact of such activity on the animals to ensure that the conservation value of primate tourism is justified. Indeed, a large number of studies indicate that tourism activity could have a negative influence on primate health status, diet and activity budget, increasing the risk of disease transmission and poaching [59,60].

The impact of tourism activity on the health status of Barbary macaques in Algeria has previously been assessed by a few studies. Firstly, evidence suggests that tourism promotes proximity and physical contact between humans and macaques, increasing the risk of transmission of zoonotic pathogens [59,60,61,62]. Indeed, several zoopathogenic agents have been detected in Barbary macaques in tourist areas in Algeria, including: five bacteria, eight protozoa, nine helminths, and one ectoparasitic insect. Furthermore, Bachiri et al. [63] found a significant prevalence of the CTX-M-15 gene in *E. coli* isolated from Barbary macaques, suggesting wide spread resistance to betalactam antibiotic among this species. Finally, a recent study showed a significant alteration in the faecal microbiota of a tourist-provisioned group of Barbary macaques [64]. This alteration may be caused by the consumption of anthropogenic foods—potentially poor in fiber and rich in simple sugars and fats—and can have negative repercussions on their health status [64]. Finally, the habituation of macaques to human presence facilitates poaching. Young individuals of anthropogenic groups are particularly targeted by poachers due to their ease of capture [48,54].

## 4. Discussion and Conservation Perspectives

This review provides the first Algeria- focused comprehensive synthesis of available knowledge on the Barbary macaque (*Macaca sylvanus*), integrating both peer-reviewed studies, thesis and institutional reports. Through this analysis, we suggest updated population estimates, an overview of current distribution, and a critical evaluation of anthropogenic threats affecting the species in Algeria.

The review reveals that Algeria may currently host over 9000 individuals, representing a significant proportion of the global population of *M. sylvanus*. This estimate is significantly higher than the figures reported by Fa et al. [1], who estimated the Algerian population to range between 4475 and 6200 individuals. However, this difference likely reflects better census efforts in recent years. Potential declines or local extinctions cannot be excluded given increasing anthropogenic pressures in the area where recent data are not available. The preliminary population estimates presented in this review should be interpreted with caution, as they are derived from diverse studies with varying methodologies. Further field investigations are essential to refine national population assessments, particularly surveys employing line transect or camera trap distance sampling techniques over broader spatial scales, as well as occupancy modelling [33]. Finally, the presence of large groups in national parks such as Djurdjura, Chréa, and Gouraya, constitutes a strategic advantage, as it facilitates the implementation of conservation programs. The Barbary macaque in Algeria is increasingly affected by a variety of anthropogenic pressures that compromise its survival and in turn the integrity of its forest habitats. These threats are often interrelated, acting synergistically to reduce habitat quality, fragment populations, increase mortality, and disrupt social and physiological functioning.

The present review has revealed critical data deficiencies and research gaps. Firstly, we note the absence of a recent population censuses in several areas, including the Akfadou and Guerrouche forests, Babors-Tababort National Park, and the Kherrata region. Secondly, there is a lack of behavioral and ecological studies on macaque foraging behavior, population dynamics and demographics, or inter-group interactions in Algeria. Thirdly, no published data on the human–macaque conflict in Algeria currently exists. Finally, there is insufficient health surveillance, despite known zoonotic transmission risks in tourist zones.

### 4.1. Conservation Recommendations

Addressing the current gaps in knowledge and protection of *M. sylvanus* in Algeria requires the development of a national research and conservation agenda, supported by academic institutions, environmental authorities, and local communities. Below we list recommendations and priorities that could help promote the conservation of *M. sylvanus* in Algeria.

First, the reinforcement of existing legislation for the protection of the Barbary macaque could contribute to reducing poaching and illegal trade.

Second, protected areas serve as essential refuges for the Barbary macaque. In addition to existing national parks (e.g., Djurdjura, Gouraya, Chréa, and Taza), other key habitats like the Akfadou and Guerrouche forests should be prioritized for formal protection. Effective conservation within these areas requires concrete management actions such as habitat restoration, monitoring programs, awareness campaigns about the impacts of provisioning, and community-based strategies to mitigate human–macaque conflicts.

Thirdly, it seems essential to implement concrete and specific measures within and outside protected areas in order to mitigate anthropogenic threats. These actions could target the reduction of roadkill through signage and speed bumps in key zones, control illegal trade (especially online), and regulate tourism activities that alter macaque behavior and health [65]. Support for local communities, including compensation schemes and alternative income-generating activities (e.g., apiculture), should be part of an inclusive conservation framework [66].

The development and implementation of appropriate conservation measures require a solid scientific basis. In this context, promoting primatological research in Algeria could provide essential data to inform adaptive management and policy decisions. Establishing academic programs and dedicated research groups may facilitate such efforts.

Finally, inspired by successful examples such as the Barbary Macaque Awareness and Conservation (BMAC) initiative in Morocco, the establishment of a national NGO focused on *M. sylvanus* conservation could help enhance citizen involvement, education, and local conservation initiatives.

### 4.2. Conclusions

The conservation of the Barbary macaque presents a complex, multifaceted challenge that requires collaboration among a variety of stakeholders, including local communities, government authorities, researchers, and non-governmental organizations. This review provides a comprehensive summary of the current scientific understanding of the Barbary macaque in Algeria, emphasizing the need for a more diversified approach in terms of geography and research themes.

## Figures and Tables

**Table 1:** Summary of field survey efforts and sampling protocols used in recent Barbary macaque population estimates across different regions of Algeria, as included in the present review.

Ref	Location	Method	No. of Observers	No. of Observations/Group	Survey Duration
[27]	N.P Taza	Direct count	2–3	8 obs.	3 months
[28]	N.P Gouraya	Direct count	2	12 obs. (2obs./day for 3 consecutive days, twice a year)	1 year
[29]	Jijel	Direct count	2	4–6 obs.	2 months
[30]	Bejaia, Jijel and Skikda	Direct count	2	2 obs.	Not specified
[31]	N.P Djurdjura	Direct count	3	9 obs.	2 years

**Table 2:** Summary of population estimates for the Barbary macaque (*Macaca sylvanus*) in Algeria based on recent censuses (2012–2023) and historical estimates where updated data are lacking. Numbers in italics with (*) represent estimates derived from older sources (Fa et al. [1]) and should be interpreted with caution.

Areas	Wilaya	Localities	Population Estimates Total		Sources
**NP of** **Djurdjura**	Tizi- Ouazou and Bouira	- Aït Ouabane	2081	**4645**	[31]
		- Tikjda	699		
		- Tala Guilef	739		
		- Tala Rana	1010		
		- Tirouda	116		
**NP of Taza**	Jijel	- Ziama Mansouria and Aouana	242	**1742**	[27]
		- Guerrouche	1500 *		[1]
**NP of Chréa**	Blida and Medea	- Hamdania (Chiffa, Tamezguida, Oued El Merdja) - Chréa - Hammam melouan	**630**		[35]
**NP of** **Gouraya**	Béjaia	/	**315**		[28]
**NP of Babor-Tababort**	Sétif, Béjaia and Jijel	/		300 *	[1]
**Populations outside ofnational** **parks**	Béjaia and Tizi-Ouazou	- Akfadou (Under classification as national park)		1310–2100 *	[1]
	Jijel	- Sidi Maarouf	13	**384**	[30]
		- Ain Lehmame	100		
		- Bordj Tahar	16		[29]
		- Al Ancer	105		
		- Khiri Oued Adjoul	51		
		- El Milia	99		
	Skikda	- Salah Bouchaour, Oued Zhour, Zitouna (Collo), Tamanart		**43**	[30]
	Béjaia	- Bourbaatache		**30**	
		- Kherrata		200 *	[1]
**Total**			**9599–10,389**		

NP = National Park.

## Data Availability

Further information on the data included in this study is available from the corresponding author upon reasonable request.

## References

[1] Fa J.E., Taub D.M., Menard N., Stewart P.J., Fa J.E. (1984). The Distribution and Current Status of the Barbary Macaque in North Africa. The Barbary Macaque.

[2] Sengupta A., Gazagne E., Albert-Daviaud A., Tsuji Y., Radhakrishna S. (2020). Reliability of Macaques as Seed Dispersers. Am. J. Primatol..

[3] Fa J.E. (1984). Habitat Distribution and Habitat Preference in Barbary Macaques (*Macaca sylvanus*). Int. J. Primatol..

[4] Taub D.M. (1977). Geographic Distribution and Habitat Diversity of the Barbary Macaque *Macaca sylvanus* L.. Folia Primatol..

[5] Ménard N. (2002). Ecological Plasticity of Barbary Macaques (*Macaca sylvanus*). Evol. Anthropol..

[6] Ménard N. (1985). Le Régime Alimentaire de *Macaca sylvanus* Dans Différents Habitats d’Algérie: I. Régime En Chênaie Décidue. Rev. Ecol. (Terre Vie).

[7] Ménard N., Vallet D. (1986). Le Régime Alimentaire de *Macaca sylvanus* Dans Différents Habitats d’Algérie: II. Régime En Forêt Sempervirente et Sur Les Sommets Rocheux. Rev. Ecol. (Terre Vie).

[8] Ménard N., Vallet D. (1993). Population Dynamics of *Macaca sylvanus* in Algeria: An 8-Year Study. Am. J. Primatol..

[9] Maibeche Y., Moali A., Yahi N., Menard N. (2015). Is Diet Flexibility an Adaptive Life Trait for Relictual and Peri-Urban Populations of the Endangered Primate *Macaca sylvanus*?. PLoS ONE.

[10] Young C., Schülke A., Ostner J., Majolo B. (2012). Consumption of Unusual Prey Items in the Barbary Macaque (*Macaca sylvanus*). Afr. Primates.

[11] Adams M.J., Majolo B., Ostner J., Schülke O., De Marco A., Thierry B., Engelhardt A., Widdig A., Gerald M.S., Weiss A. (2015). Personality Structure and Social Style in Macaques. J. Pers. Soc. Psychol..

[12] Thierry B. (2007). Unity in Diversity: Lessons from Macaque Societies. Evol. Anthropol..

[13] Fa J.E. (1983). The Barbary Macaque. Oryx.

[14] Morales J.C., Melnick D.J. (1998). Phylogenetic Relationships of the Macaques (Cercopithecidae: Macaca), as Revealed by High Resolution Restriction Site Mapping of Mitochondrial Ribosomal Genes. J. Hum. Evol..

[15] Fooden J. (2007). Systematic Review of the Barbary Macaque, *Macaca sylvanus* (Linnaeus, 1758). Fieldiana Zool..

[16] Mehlman P. (1986). Male Intergroup Mobility in a Wild Population of the Barbary Macaque (*Macaca sylvanus*), Ghomaran Rif Mountains, Morocco. Am. J. Primatol..

[17] Scheffrahn W., Ménard N., Vallet D., Gaci B. (1993). Ecology, Demography, and Population Genetics of Barbary Macaques in Algeria. Primates.

[18] Von Segesser F., Menard N., Gaci B., Martin R.D. (1999). Genetic Differentiation within and between Isolated Algerian Subpopulations of Barbary Macaques (*Macaca sylvanus*): Evidence from Microsatellites. Mol. Ecol..

[19] Ménard N., Rantier Y., Foulquier A., Qarro M., Chillasse L., Vallet D., Pierre J.S., Butet A. (2014). Impact of Human Pressure and Forest Fragmentation on the Endangered Barbary Macaque *Macaca sylvanus* in the Middle Atlas of Morocco. Oryx.

[20] Ciani A.C., Palentini L., Arahou M., Martinoli L., Capiluppi C., Mouna M. (2005). Population Decline of *Macaca sylvanus* in the Middle Atlas of Morocco. Biol. Conserv..

[21] Butynski T.M., Cortes J., Waters S., Fa J., Hobbelink M.E., van Lavieren E., Belbachir F., Cuzin F., de Smet K., Mouna M. (2008). Macaca sylvanus, The IUCN Red List of Threatened Species: T12561A335.

[22] Wallis J., Pilot M., Majolo B., Waters S.S. (2020). Macaca sylvanus. TheIUCN Red List of Threatened Species 2020: E.T12561A50043570.

[23] Estrada A., Garber P.A., Rylands A.B., Roos C., Fernandez-Duque E., Di Fiore A., Anne-Isola Nekaris K., Nijman V., Heymann E.W., Lambert J.E. (2017). Impending Extinction Crisis of the World’s Primates: Why Primates Matter. Sci. Adv..

[24] Majolo B., van Lavieren E., Maréchal L., MacLarnon A., Marvin G., Qarro M., Semple S., Radhakrishna S., Huffman M., Sinha A. (2013). Out of Asia: The Singular Case of the Barbary Macaque. The Macaque Connection.

[25] El Alami A., Van Lavieren E., Ahmim M., Namous S., Fattah A., Znari M., Chait A. (2021). Distribution, Population Status and Ecology of the Endangered Barbary Macaque *Macaca sylvanus* in North Africa. Int. J. Sci. Res. Biol. Sci..

[26] El Alami A., Van Lavieren E., Chait A. (2022). The Major Threats To The Endangered Barbary Macaque *Macaca sylvanus* In North Africa. Eur. J. Ecol..

[27] Bengana M.-N., Ziani D., Mousli M. (2018). Bio-Ecologie Du Magot (Macaca sylvanus L.) Dans Le Parc Arc National De Taza (Wilaya de Taza (Wilaya de Jijel).

[28] National Park of Gouraya (2016). Etude D’inventaire des Troupes de Singes Dans le Parc National de Gouraya.

[29] Soukkou N., Bensaada N., F A R Z. (2023). Etude Écologique et Comportementale Du Singe Magot *Macaca sylvanus* (Linné, 1758) Dans La Région de Jijel. Master’s Thesis.

[30] Ahmim M., Labiod A. (2020). New Data on the Current Distribution of Barbary Macaque *Macaca sylvanus* (Mammalia: Cercopithecidae) in Algeria. Am. J. Life Sci..

[31] National Park of Djurdjura (2021). Étude D’inventaire des Troupes de Singes Dans Le Parc National Du Djurdjura: Population, Distribution et Plan d’action.

[32] QGIS Development Team (2024). QGIS Desktop v3.36.0 User Guide.

[33] Plumptre A.J., Sterling E.J., Buckland S.T., Sterling E., Bynum N., Blair M. (2013). Primate Census and Survey Techniques. Primate Ecology and Conservation.

[34] Kerbiche F., Aknine Souidi R. (2023). The Management of National Parks in Algeria: Overview, Means and Constraints. Case of National Parks in the North. J. Econ. Sustain. Dev..

[35] National Park of Chrea (2020). Plan de Gestion Du Parc National de Chréa (Période Quinquennal 2020/2024).

[36] National Park of Taza (2020). Plan de Gestion Du Parc National de Taza (2020–2024).

[37] Zerouali B., Santos C.A.G., do Nascimento T.V.M., da Silva R.M. (2023). A Cloud-Integrated GIS for Forest Cover Loss and Land Use Change Monitoring Using Statistical Methods and Geospatial Technology over Northern Algeria. J. Environ. Manage..

[38] Aini A., Curt T., Bekdouche F. (2019). Modelling Fire Hazard in the Southern Mediterranean Fire Rim (Bejaia Region, Northern Algeria). Environ. Monit. Assess..

[39] Bosse M., van Loon S. (2022). Challenges in Quantifying Genome Erosion for Conservation. Front. Genet..

[40] Modolo L., Salzburger W., Martin R.D. (2005). Phylogeography of Barbary Macaques (*Macaca sylvanus*) and the Origin of the Gibraltar Colony. Proc. Natl. Acad. Sci. USA.

[41] Martínez Sosa F., Benrabah M.E., Majolo B., Pilot M. (2022). Genetic Diversity of Barbary Macaques (*Macaca sylvanus*) and Its Implications in Conservation Management of the Species. Biol. Life Sci. Forum.

[42] Le Gouar P., Vallet D., Ernoult A., Petit E.J., Rantier Y., Dréano S., Qarro M., Ménard N. (2021). A Multiscale Analysis of Landscape Resistance Reveals Genetic Isolates in an Endangered Forest-Specialist Species the Barbary Macaque (*Macaca sylvanus*). Biol. Conserv..

[43] Ciani A.C., Martinoli L., Capiluppi C., Arahou M., Mouna M. (2001). Effects of Water Availability Habitat Quality on Bark-Stripping Behavior in Barbary Macaques. Conserv. Biol..

[44] Fa J.E. (1986). On the Ecological Status of the Barbary Macaque *Macaca sylvanus* L. in North Morocco: Habitat Influences versus Human Impact. Biol. Conserv..

[45] Ménard N., Motsch P., Delahaye A., Saintvanne A., Le Flohic G., Dupé S., Vallet D., Qarro M., Tattou M.I., Pierre J.-S. (2014). Effect of Habitat Quality on Diet Flexibility in Barbary Macaques. Am. J. Primatol..

[46] Van Lavieren E., Wich S.A. (2010). Decline of the Endangered Barbary Macaque *Macaca sylvanus* in the Cedar Forest of the Middle Atlas Mountains, Morocco. Oryx.

[47] Ménard N., Vallet D., Gautier-Hion A. (1985). Demography and reproduction of *Macaca sylvanus* in different habitats in Algeria. Folia Primatol..

[48] Ménard N., Foulquier A., Vallet D., Qarro M., Le Gouar P., Pierre J.S. (2014). How Tourism and Pastoralism Influence Population Demographic Changes in a Threatened Large Mammal Species. Anim. Conserv..

[49] Oudahmane M., Moali A., Bekdouche F. (2018). Effects of Human Pressure on the Distribution of *Macaca sylvanus* in the Sector of Ait Ouabane, National Park of the Djurdjura, Algeria. Ecol. Environ. Conserv..

[50] Hughes J.D. (2003). Europe as Consumer of Exotic Biodiversity: Greek and Roman Times. Landsc. Res..

[51] Van Uhm D. (2016). Monkey Business: The Illegal Trade in Barbary Macaques. J. Traffick. Organ. Crime Secur..

[52] Jaap G., Brandon- Jones D. (1999). Mumies of Olive Baboons and Barbary Macaques in the Baboon Catacomb of the Sacred Animal Necropolis at North Saqqara. J. Egypt. Archaeol..

[53] Bailey J.F., Henneberg M., Colson I.B., Ciarallo A., Hedges R.E.M., Sykes B. (1999). Monkey Business in Pompeii—Unique Find of a Juvenile Barbary Macaque Skeleton in Pompeii Identified Using Osteology and Ancient DNA Techniques. Mol. Biol. Evol..

[54] Van Lavieren E. (2004). The Illegal Trade in the Moroccan Barbary Macaque (*Macaca sylvanus*) and the Impact on the Wild Population. Master’s Thesis.

[55] Bergin D., Atoussi S., Waters S. (2018). Online Trade of Barbary Macaques *Macaca sylvanus* in Algeria and Morocco. Biodivers. Conserv..

[56] Atoussi S., Razkallah I., Ameziane I.N., Boudebbouz A., Bara M., Bouslama Z., Houhamdi M. (2022). Illegal Wildlife Trade in Algeria, Insight via Online Selling Platforms. Afr. J. Ecol..

[57] Benkacimi S., Bendjedda N. (2015). Evaluation Des Effets de La Surfréquentation Touristique Sur La Viabilité Du Singe Magot (*Macaca sylvanus*) Dans La Région de La Chiffa et Proposition d’Un Modèle d’Écotourisme Durable (Parc National de Chréa). Master’s Thesis.

[58] Mallapur A., Radhakrish-na S., Huffman M.A., Sinha A. (2013). Macaque Tourism: Implications for Their Management and Conservation. The Macaque Connection. Cooperation and Conflict Between Humans and Macaques.

[59] Russon A.E. (2014). Wallis, J. Primate Tourism: A Tool for Conservation?.

[60] Muehlenbein M.P., Wallis J. (2014). Considering Risks of Pathogen Transmission Associated with Primate-Based Tourism. Primate Tourism: A Tool for Conservation.

[61] Patouillat L., Hambuckers A., Adi Subrata S., Garigliany M., Brotcorne F. (2024). Zoonotic Pathogens in Wild Asian Primates: A Systematic Review Highlighting Research Gaps. Front. Vet. Sci..

[62] Carne C., Semple S., MacLarnon A., Majolo B., Maréchal L. (2017). Implications of Tourist–Macaque Interactions for Disease Transmission. Ecohealth.

[63] Bachiri T., Bakour S., Ladjouzi R., Thongpan L., Rolain J.M., Touati A. (2017). High Rates of CTX-M-15-Producing Escherichia Coli and Klebsiella Pneumoniae in Wild Boars and Barbary Macaques in Algeria. J. Glob. Antimicrob. Resist..

[64] Boumenir M., Hornick J.-L., Taminiau B., Daube G., Brotcorne F., Iguer-Ouada M., Moula N. (2022). First Descriptive Analysis of the Faecal Microbiota of Wild and Anthropized Barbary Macaques (*Macaca sylvanus*) in the Region of Bejaia, Northeast Algeria. Biology.

[65] Maréchal L., Semple S., Majolo B., MacLarnon A. (2016). Assessing the Effects of Tourist Provisioning on the Health of Wild Barbary Macaques in Morocco. PLoS ONE.

[66] Dawson N.M., Coolsaet B., Bhardwaj A., Booker F., Brown D., Lliso B., Loos J., Martin A., Oliva M., Pascual U. (2024). Is It Just Conservation? A Typology of Indigenous Peoples’ and Local Communities’ Roles in Conserving Biodiversity. One Earth.

